# Augmin complex activity finetunes dendrite morphology through non-centrosomal microtubule nucleation *in vivo*

**DOI:** 10.1242/jcs.261512

**Published:** 2024-05-10

**Authors:** Yun Zhang, Hsin-Ho Sung, Anna B. Ziegler, Ying-Chieh Wu, Ricardo Viais, Carlos Sánchez-Huertas, Lukas Kilo, Fikret Gürkan Agircan, Ying-Ju Cheng, Kousuke Mouri, Tadashi Uemura, Jens Lüders, Cheng-Ting Chien, Gaia Tavosanis

**Affiliations:** ^1^ German Center for Neurodegenerative Diseases (DZNE), Dynamics of Neuronal Circuits Group, Venusberg Campus 1 Building 99, 53127 Bonn, Germany; ^2^ Institute of Molecular Biology, Academia Sinica, 11529 Taipei, Taiwan; ^3^ Institute for Research in Biomedicine (IRB Barcelona), The Barcelona Institute of Science and Technology, Baldiri Reixac 10, 08028 Barcelona, Spain; ^4^ Graduate School of Biostudies, Kyoto University, Kyoto 606-8501, Japan; ^5^ Center for Living Systems Information Science, Kyoto University; ^6^ LIMES Institute, University of Bonn, 53115 Bonn, Germany

**Keywords:** Neuronal dendrites, Microtubules, Augmin, HAUS, γ-tubulin, *Drosophila* c4da neurons, Hippocampal neurons

## Abstract

During development, neurons achieve a stereotyped neuron type-specific morphology, which relies on dynamic support by microtubules (MTs). An important player is the augmin complex (hereafter augmin), which binds to existing MT filaments and recruits the γ-tubulin ring complex (γ-TuRC), to form branched MTs. In cultured neurons, augmin is important for neurite formation. However, little is known about the role of augmin during neurite formation *in vivo*. Here, we have revisited the role of mammalian augmin in culture and then turned towards the class four *Drosophila* dendritic arborization (c4da) neurons. We show that MT density is maintained through augmin in cooperation with the γ-TuRC *in vivo*. Mutant c4da neurons show a reduction of newly emerging higher-order dendritic branches and in turn also a reduced number of their characteristic space-filling higher-order branchlets. Taken together, our data reveal a cooperative function for augmin with the γ-TuRC in forming enough MTs needed for the appropriate differentiation of morphologically complex dendrites *in vivo*.

## Teaser

**Highlighted Article:** During neuronal development augmin stimulates microtubule nucleation and is required *in vivo* in *Drosophila* c4da neurons to fine-tune their complex dendrite morphology.

## INTRODUCTION

**Fig. 1.**
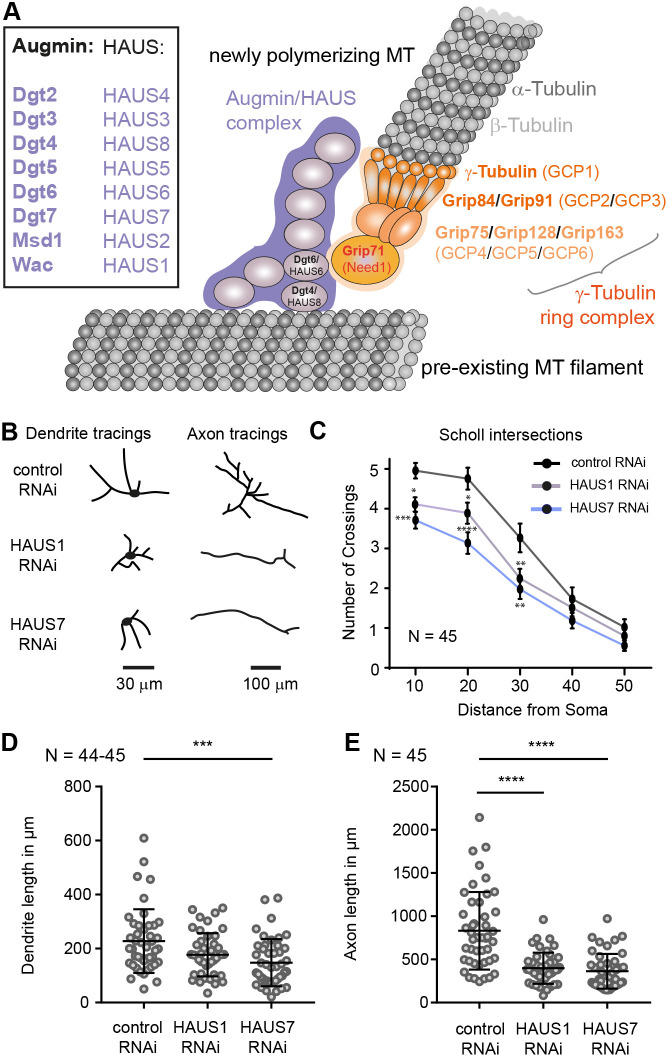
**Depletion of HAUS1 or HAUS7 complex members impairs dendrite and axon growth of hippocampal neurons.** (A) Augmin consists of eight subunits (Dgt2–7, Wac and Msd1 in *Drosophila* and HAUS1–HAUS8 in mammals) and interacts via Dgt4 (the homolog of HAUS8) with pre-existing MT polymers. The γ-TuRC (orange) is recruited by an interaction between Dgt6 (the homolog of HAUS6) and Grip71 (the homolog of Need1). See text for further details. (B–E) Primary hippocampal neuron cultures were transfected with shRNA- and GFP-expressing plasmids at 1DIV and fixed at 4DIV. (B) Representative tracings of cultured hippocampal neurons expressing a control plasmid (expressing a scrambled sequence), HAUS1 or HAUS7 RNAi constructs. (C) Depletion of HAUS1 or HAUS7 reduced neurite complexity as measured by Scholl analysis. Error bars are mean±s.e.m. (D,E) Quantification of total dendritic and total axonal length of neurons as shown in B; mean±s.d. ***P≤0.001; ****P≤0.0001 (Kruskal–Wallis with Dunn's multiple comparison test). *N* reflects number of neurons (biological replicates) from three experiments.

Neurons are highly polarized cells that display a high morphological variability (Neukirchen and Bradke, 2011; Hill et al., 2012; Lefebvre et al., 2015). To a large extent, this variation is caused by the diversity of the neuron type-specific dendritic trees (Jan and Jan, 2010; Lefebvre et al., 2015; Tavosanis, 2021). The branched dendrites receive and integrate input information; therefore, their complexity relates to the number and distribution of their inputs. Neuron type-specific dendritic morphologies are established during development and any changes leading to errors during this process can impact the function of the mature neuron (Ziegler et al., 2017; Ferreira Castro et al., 2020). The cytoskeleton is essential for dendrite elaboration. Although actin is involved in the dynamics that support dendrite branching and elaboration, microtubules (MTs) are thought to promote the stabilization of branch subsets (Delandre et al., 2016; Nanda et al., 2020; Kilo et al., 2021). MTs are polarized polymers nucleating from microtubule-organizing centers (MTOCs) (González et al., 1998; Sanchez and Feldman, 2017; Wu and Akhmanova, 2017). A key component of MTOCs is γ-tubulin (γ-Tub), which assembles with the γ-Tub complex proteins (GCPs) into the γ-Tub ring complex (γ-TuRC). In *Drosophila melanogaster*, the GCPs include Grip75, Grip84, Grip91, Grip128 and Grip163; in mammals they are termed GCP2–GCP6 (GCP2–GCP6 are also known as TUBGCP2–TUBGCP6) (Fig. 1) (Gunawardane et al., 2000; Thawani and Petry, 2021). In proliferating cells γ-TuRCs concentrate at the centrosome, which functions as the major MTOC (González et al., 1998). However, during differentiation, centrosomes of rodent and fly neurons gradually lose γ-Tub and, concurrently, MTOC activity, while γ-Tub localization is shifted to the cytoplasm, suggesting that the role of the centrosome is taken over by acentrosomal MT nucleation mechanisms in post-mitotic neurons (Leask et al., 1997; Stiess et al., 2010; Yonezawa et al., 2015; Sánchez-Huertas et al., 2016; Vinopal et al., 2023). In fact, reducing this non-centrosomal γ-Tub fraction has been shown to lead to decreased MT polymerization in later stage neurons resulting in reduced neurite number and length (Sánchez-Huertas et al., 2016; Cunha-Ferreira et al., 2018). Multiple studies have thus focused on clarifying the mechanisms behind γ-Tub-dependent cytosolic MT polymerization in post-mitotic neurons. In the soma of differentiated *Drosophila* dendritic arborization (da) neurons, fluorescently labeled γ-Tub localizes predominantly to the Golgi stacks, and Golgi outposts in proximal dendrites were suggested to gain MTOC activity (Ori-McKenney et al., 2012; Wu and Akhmanova, 2017; Mukherjee et al., 2020). However, follow up studies indicate that γ-Tub-dependent MT nucleation in dendrites can also work independently of an interaction with Golgi outposts (Nguyen et al., 2014; Yalgin et al., 2015; Mukherjee et al., 2020). Along this line, very recently, endosomes at the growing tips of dendrites were additionally described as MT nucleation sites in *Caenorhabditis elegans* and *Drosophila* dendrites (Liang et al., 2020; Weiner et al., 2020).

As an alternative to organelle-based MTOC activity, MTs can also form from pre-existing MT filaments. In such a scenario, short MT fragments could act as seeds that provide free plus-ends for MT elongation. These MT seeds might be created through severing of existing filaments and minus-end stabilization (Wood et al., 2006; Yu et al., 2008; Stewart et al., 2012; Jiang et al., 2014; Buijs et al., 2021). This model is supported by observations made in fruit fly c4da neurons mutant for the MT-severing ATPases Spastin and Katanin 60L1 which show reduced dendritic complexity (Stewart et al., 2012; Buijs et al., 2021). Another possibility is that MTs are nucleated as branches from pre-existing MTs by the augmin complex (hereafter augmin). This hetero-octameric complex was first shown to recruit γ-TuRCs to the lattice of a pre-existing mother MT filament and thereby trigger new MT growth within the mitotic spindle in dividing *Drosophila* Schneider 2 cells (Goshima et al., 2008). Augmin and its mammalian homologa, the homologous to augmin subunit (HAUS) complex, consist of eight subunits, termed Dgt2–Dgt7 (Dgt7 is also known as Msd5), Wac and Msd1 in *Drosophila* and HAUS1–HUAS8 in mammals (Fig. 1A) (Wu et al., 2008; Lawo et al., 2009; Uehara et al., 2009; Hsia et al., 2014). Recent structural studies have shown that augmin is composed of a V-shaped head made of Msd1, Dgt4, Dgt6 and Dgt7, which are the equivalent of HAUS2, HAUS8, HAUS6 and HAUS7 in mammals, and a tail made of Dgt2, Dgt3, Dgt5, and Wac, which are the equivalent of HAUS4, HAUS3, HAUS5 and HAUS1 in mammals (Hsia et al., 2014; Gabel et al., 2022). All eight subunits are required for augmin to fulfill its function. However, augmin subunit HAUS8/Dgt4 is primarily responsible for binding to the MT lattice, whereas γ-TuRC is recruited through an interaction between Dgt6 (HAUS6 in mammals) and Grip71 (Nedd1 in mammals) to set a starting point for a newly nucleating MT branch (Wu et al., 2008; Lawo et al., 2009; Uehara et al., 2009; Hsia et al., 2014; Song et al., 2018). *In vitro*, purified augmin alone does not change MT nucleation dynamics, whereas branched MT density was significantly enhanced upon the cooperation of augmin and purified γ-TuRC complexes, indicating that augmin strictly requires the γ-TuRC for MT-based nucleation (Alfaro-Aco et al., 2020; Tariq et al., 2020).

A recent set of studies has revealed the importance of augmin function in dendrite and axon development of rodent hippocampal neurons (Sánchez-Huertas et al., 2016; Cunha-Ferreira et al., 2018; Viais et al., 2021). In detail, during neocortical development, augmin is at first necessary for initial neuronal polarization and radial migration in embryonic mouse neurons *in vivo* (Cunha-Ferreira et al., 2018). At a later developmental stage, knockdown of augmin complex members or of γ-Tub in cultured hippocampal neurons decreases neurite number, length and complexity (Sánchez-Huertas et al., 2016; Cunha-Ferreira et al., 2018). In fact, reduced expression of augmin complex members phenocopies the morphological simplification observed in γ-Tub knockdown neurons (Sánchez-Huertas et al., 2016; Cunha-Ferreira et al., 2018). This phenotype can be rescued by simultaneous overexpression of HAUS subunits (Cunha-Ferreira et al., 2018). Augmin and γ-TuRC interact biochemically, as HAUS6 can be co-immunoprecipitated with the γ-TuRC member GCP3 in lysates from cultured hippocampal cells *in vitro* (Sánchez-Huertas et al., 2016). Additionally, GFP-tagged HAUS2 has been shown to colocalize by ∼45% with mCherry-tagged γ-TuRC complex member GCP2 at 10 days *in vitro* (DIV10). Finally, p-Syn-tdTomato-MACF18, a MT plus-end marker, labeled MT plus-end tips emerging from GFP–HAUS2-labeled clusters indicating that MTs can be nucleated in an augmin-dependent manner (Cunha-Ferreira et al., 2018). In consequence, loss of augmin subunits leads to a decreased number of polymerized MTs (Sánchez-Huertas et al., 2016; Cunha-Ferreira et al., 2018). Taken together, these studies show that augmin interacts with the γ-TuRC to nucleate MTs in neurites and support neurite morphogenesis.

In addition to this role in dendrite and axonal growth, loss of augmin also affects MT polarity in axons. MTs in axons are oriented with their fast-growing plus-end pointing away from the cell body and thus polymerize in the anterograde direction. By contrast, although very early generated dendrites are marked by high levels of plus-end-out MTs, during later differentiation minus-end-out MTs are gradually added until a mixed polarity is obtained in vertebrate dendrites and an almost uniform minus-end-out polarity is obtained in invertebrate dendrites (Stone et al., 2008; Hill et al., 2012; Yau et al., 2016; Feng et al., 2019). Therefore, in invertebrate dendrites, MTs are mostly polymerizing in retrograde direction (towards the cell body). Multiple mechanisms have already been identified that control MT polarity in axons and dendrites (recently reviewed by Rolls, 2022). In cultured neurons, knocking down augmin subunits increases the fraction of retrograde-polymerizing MTs in axons leading to axons with MTs of mixed polarity. This led to the hypothesis that augmin-mediated MT nucleation is used to produce new MTs based on the polarity of the pre-existing MTs (Sánchez-Huertas et al., 2016; Cunha-Ferreira et al., 2018). By contrast, MT polarity has been shown to be unaffected by loss of augmin function in dendrites of cultured neurons (Cunha-Ferreira et al., 2018). Finally, stage embryonic day (E)13.5 and E17.5 embryos of a conditional mouse HAUS6-knockout (KO) mutant display massive defects in brain development. For example, the radial thickness of the neuronal layer within the thalamus is reduced by 90%. However, those defects were linked to mitotic errors as well as p53-dependent apoptosis, and no effects of HAUS6 loss on neurons after E17.5 were examined (Viais et al., 2021).

Taken together, these studies point towards an important role of augmin in γ-TuRC-dependent MT nucleation and morphogenesis in neurons. However, although knockdown of augmin subunits have been shown to impair dendritic complexity in mature neurons in culture, the role of augmin in dendrite formation during early developmental stages and how it affects dendrite development *in vivo* has not been explored.

Larval *Drosophila* dendritic arborization (da) neurons have been extensively used for *in vivo* studies on the neurodevelopmental role of cytoskeletal regulators (Jan and Jan, 2010). The da sensory neurons extend their dendrites under the almost transparent cuticle of the animals. Da neurons are classified into four classes based on the complexity of their dendritic trees and their distinct functions, with c1da proprioceptive neurons displaying a simple dendritic tree and c4da neurons, which respond to nociceptive stimuli, displaying a highly elaborate arbor (Grueber et al., 2002; Hughes and Thomas, 2007; Hwang et al., 2007).

In this study, we first revisited the role of the augmin in cultured mouse hippocampal neurons and found that it is important for developmental growth of early-stage dendrites. Knockdown of two different augmin subunits did not alter MT polarity but reduced dendritic microtubule growth and density. To determine how branching nucleation affected dendritic development *in vivo*, we next analyzed the function of the *Drosophila* homolog of the augmin complex in supporting dendrite arborization in the c4da neurons. Here, in agreement with the findings in cultured rodent neurons, we first provide evidence for reduced MT density along dendritic projections in augmin mutant neurons compared to what is seen for controls. Using *in vivo* time-lapse imaging, we show that this loss in MT density correlated with a reduced number of newly forming dendritic higher order branches, which are the morphological hallmarks of c4da neurons (Grueber et al., 2002). Finally, the genetic interaction of augmin and γ-TuRC suggests a functional relationship in this neurodevelopmental process *in vivo*. Taken together, using neuronal culture and *in vivo* models, our data provide evidence for a coordinated action of augmin and the γ-TuRC to support non-centrosomal MT nucleation in developing dendrites, which is required for the formation of higher-order dendritic branches in morphologically complex neurons. Together with previous work, our results establish augmin as a crucial factor for non-centrosomal microtubule nucleation across all neuronal compartments and developmental stages to drive neuronal morphogenesis.

## RESULTS

### Depletion of HAUS1 or HAUS7 impairs dendrite and axonal length

During differentiation, embryonic cortical neurons initially develop a trailing process that later becomes the axon, and a leading edge that becomes the apical dendrite (Polleux and Snider, 2010). An increased fraction of mouse cortical neurons in which the augmin member HAUS6 is depleted lack these processes at embryonic stage E14.5. Also, differentiated late-stage mouse hippocampal neurons in culture [days *in vitro* (DIV)12] in which HAUS2 or HAUS6 have been knocked down have reduced dendritic complexity (Cunha-Ferreira et al., 2018). Complementing this work, we here tested the role of augmin in neurite outgrowth at very early stages by depleting two other subunits (HAUS1 or HAUS7) via RNA interference (RNAi) in cultured mouse hippocampal neurons from DIV1 to DIV4 (Fig. 1B,C). Efficient knockdown of augmin by HAUS1 or HAUS7 shRNA was demonstrated by western blotting in our previous study (Sánchez-Huertas et al., 2016). We measured reduced dendritic spanning reflected by a lower number of crossings of Scholl intersections in depleted neurons (Fig. 1C). Additionally, total dendritic length was reduced (Fig. 1D). Axon length in depleted neurons was also shorter (Fig. 1E). Thus, augmin is involved in dendrite growth not only in more mature neurons (DIV12) but also during very early differentiation stages (DIV4 and below).

**Fig. 2.**
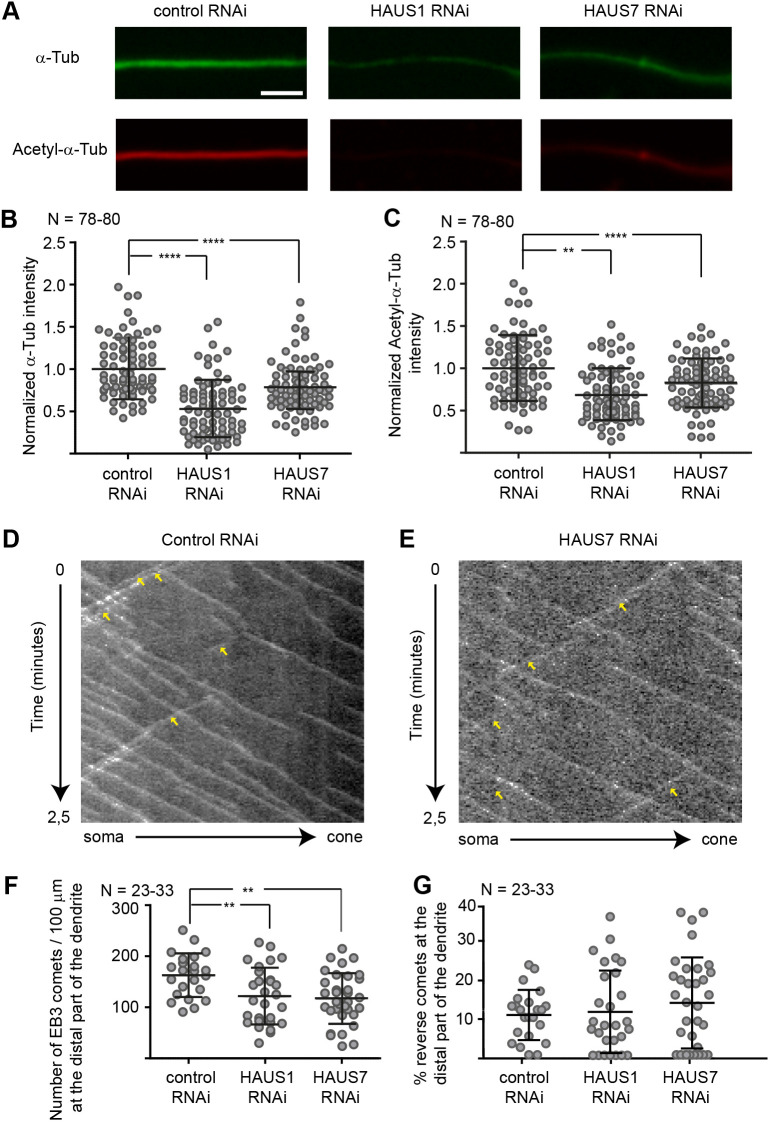
**HAUS1 or HAUS7 depletion reduces the amount of tubulin in dendrites but does not affect microtubule polarity.** (A–C) Primary hippocampal neuronal cultures were transfected with shRNA-expressing plasmids at 1DIV and fixed and stained using anti-α-Tub and anti-acetyl-α-Tub antibodies at 4DIV. (A) Representative images of α-Tub and acetyl-α-Tub labeling in dendrites. Scale bar: 10 µm. (B,C) Quantification (mean±s.d.) of the normalized mean signal intensity of α-Tub or acetyl-α-Tub in dendrites. (D–G) Primary hippocampal neuron cultures were co-transfected at 3DIV with HAUS1 or HAUS7 shRNA constructs and an EB3::tomato expression construct and imaged at 5DIV. (D,E) Representative kymographs of time-lapse recordings of EB3 comets in control or HAUS7-depleted dendrites. Reverse comets are marked by arrowheads. (F,G) Quantification (mean±s.d.) of the number of EB3-positive comets in the distal (F) part of the dendrites and percentage of retrograde comets (G) from kymographs as in D and E. **P≤0.01; ****P≤0.0001 [Kruskal–Wallis followed by Dunn's multiple comparison test (B); one-way ANOVA followed by Dunnett's multiple comparison test (C,F,G)]. *N* reflects number of neurons (biological replicates) from three experiments.

Augmin has been shown to sustain adequate levels of polymerized tubulin as the dendrites of HAUS6-depleted neurons at DIV12 display decreased levels of α-Tub and acetylated (acetyl-)α-Tub (Cunha-Ferreira et al., 2018). We here analyzed α-Tub and acetyl-α-Tub levels in developing dendrites of HAUS1- or HAUS7-depleted neurons at the earlier DIV4 stage and found them to be decreased (Fig. 2A–C). Together with previous work (Sánchez-Huertas et al., 2016; Cunha-Ferreira et al., 2018), our results suggest that augmin is involved in both establishment and maintenance of a dense MT array in dendrites of cultured neurons.

The lack of augmin function has been previously shown to affect not only MT density but also MT polarity, as developing axons of cultured HAUS1- and HAUS7-depleted mouse neurons at DIV4 contain MTs of reverse polarity compared to the almost exclusive plus-end-out orientation in control axons (Sánchez-Huertas et al., 2016). In contrast, no augmin-dependent impact on MT polarity was found in dendrites of later stage neurons (DIV12) (Sánchez-Huertas et al., 2016; Cunha-Ferreira et al., 2018). We have carefully revisited this finding by knocking down HAUS1 or HAUS7 between 1DIV to 5DIV and using EB3–Tomato, a MT plus-end-binding protein that allows tracing the direction of MT growth (Stepanova et al., 2003). No effect of HAUS1 or HAUS7 depletion could be observed in proximal dendrites, but we found a decreased total number of EB3-labeled comets in the distal segments of dendrites. However, MT polarity in distal segments was not changed (Fig. 2D–G; Fig. S1). Taken together, in agreement with previous findings in more mature neurons (Sánchez-Huertas et al., 2016; Cunha-Ferreira et al., 2018), our data show that augmin is important for nucleating MTs in developing dendrites, impacting on MT density and stability, but not on MT polarity.

### Dgt5 affects MT density in c4da neurons

**Fig. 3.**
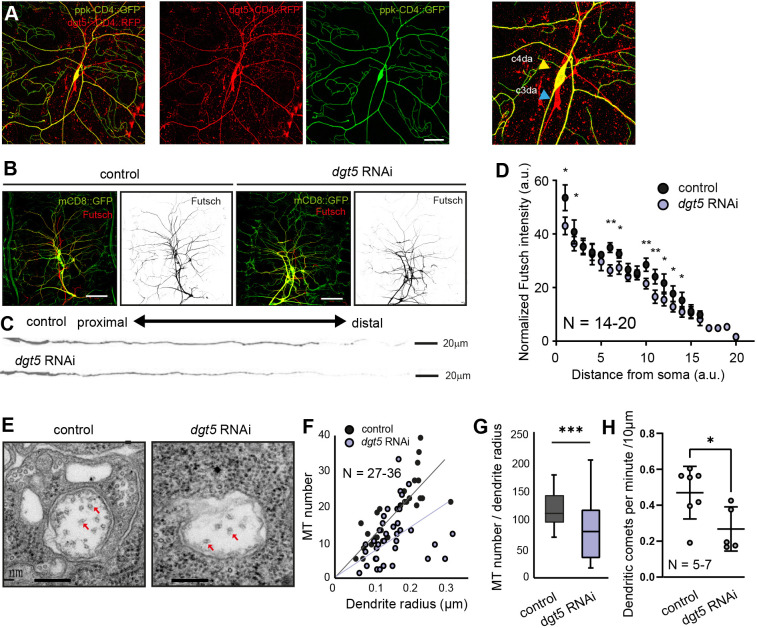
**Dgt5 supports appropriate MT density *in vivo* in da neurons.** (A) *dgt5-Gal4* mediated expression of *UAS-CD4::RFP* colocalizes with c4da marker ppk-CD4::GFP. Images representative of four repeats. Scale bar: 200 µm. (B–D) Control or *UAS-dgt5* RNAi constructs were co-expressed with *UAS-mCD::8GFP* in da neurons using *109(2)80-Gal4*. Scale bar: 100 µm. (B) Larval fillet preparations immunolabeled with anti-GFP antibodies (green; da neurons) and anti-Futsch antibodies (red; microtubules). Scale bars: 100 µm. (C) Individual dendrites were straightened by post-image processing and labeled with anti-Futsch antibodies. (D) Distribution of anti-Futsch signal intensity along the relative length of dendrites. For quantification, every dendrite was divided to 20 segments and the average Futsch signal intensity normalized to mCD8::GFP intensity was calculated for each of the segments. Error bars are mean±s.d.; a.u., arbitrary units. (E–G) MT tubule number quantification in control and *dgt5* RNAi-depleted neurites. (E) EM images showing dendrite cross-sections of a control (left panel) and a *dgt5* RNAi-depleted neurite (right panel). MTs are indicated by red arrows. Scale bars: 200 nm. (F) MT numbers divided by the dendrite radius were plotted against the dendrite radius. (G) MT number divided by dendrite radius. The box represents the 25–75th percentiles, and the median is indicated. The whiskers show the complete range. (H) Plus ends of growing microtubules are labeled by ppk-EB1::GFP and c4da neurites were identified using *ppk-Gal4* driven expression of *UAS-CD4::RFP*. EB1-comet number was measured in thin distal dendrites of control and *dgt5-RNAi* depleted cells. Error bars are mean±s.e.m. *P≤0.05; **P≤0.01; ***P≤0.001 (unpaired two-tailed Student's *t*-test). *N* reflects number of neurons (biological replicates).

We next aimed at investigating whether depleting *Drosophila* augmin would also affect MT density in dendrites *in vivo* using c4da neurons as a cellular model*.* To test whether these neurons express augmin, we used the *PBac{IT. GAL4}dgt5^0899-G4^* allele, which carries a Gal4 inserted into the *dgt5* 5′UTR and hence could represent a reporter for *dgt5* expression. *Dgt5-Gal4*-mediated expression of *UAS-CD4::RFP* colocalized with the c4da neuronal marker *ppk-CD4::GFP* (Fig. 3A). An additional neuronal cell type within the larval peripheral nervous system (PNS) was labeled, which is most likely a c3da neuron (Fig. 3A). To examine the MT levels in c4da neurons with reduced augmin complex function, we firstly labeled identified da neuronal dendrites by *109(2)80-Gal4* driven expression of membrane-tethered *UAS-mCD8GFP* and colabeled these cells using a monoclonal anti-Futsch antibody. Futsch is the fly homolog of microtubule-associated protein 1B (MAP1B). It binds MTs and its labeling intensity, detected by immunohistochemistry, can be used as a proxy for MT density (Fig. 3B) (Hummel et al., 2000). Anti-Futsch immunolabeling gradually decreased along the length of control c4da neuronal dendrites. Simultaneous expression of a *dgt5* RNAi construct, which efficiently knocked down *dgt5* expression levels (Fig. S2A,B), reduced anti-Futsch immunolabeling along the length of the dendrites (Fig. 3C,D). We verified this reduction using an alternative MT marker (Jupiter::mCherry) (Karpova et al., 2006) (Fig. S3A–C) and studying γ-Tub::GFP localization in c4da neurons (Fig. S3D–F). Note that the *ppk-Gal4* driver used for this experiment is not fully specific for c4da neurons and is additionally weakly expressed in a c3da neuron (Fig. S3D). However, the c4da neuronal dendrites could be distinguished from the c3da neuronal ones by the more specific *ppk::tdTomato* expression. γ-Tub::GFP labeling could be observed in the main branches and the fine higher-order branchlets of control c4da neurons, whereas this signal was absent or very weak in the higher-order branchlets upon *dgt5* knockdown. In contrast to this effect on MT distribution, actin localization revealed by the actin reporter LifeAct–GFP (Riedl et al., 2008) was not modified within these branchlets after RNAi-mediated knockdown of *dgt5* (Fig. S3G,H).

Finally, we quantified MT densities in electron microscopy (EM) images obtained from control da neurons and after *dgt5* knockdown (Fig. 3E–G). In our EM images, we could not assay the exact position of the cut dendrite within its neuron. Upon *dgt5* knockdown, MT density was clearly reduced in thinner dendrites, which most likely corresponds to the more-distal dendrite fragments (Fig. 3F,G). To address whether this correlates with a reduced amount of newly polymerizing MTs, we imaged EB1::GFP comets in c4da terminal dendrites and found a reduction upon *dgt5* depletion (Fig. 3H). In rodent HAUS mutant neurons in culture, the uniform MT polarity is shifted towards a mixed one (Sánchez-Huertas et al., 2016; Cunha-Ferreira et al., 2018). To test the polarity of MTs in axons *in vivo*, we additionally assayed EB1::GFP comets in c4da neuronal axons. We did not find a significant reduction of EB1::GFP comets but confirmed that, in axons, loss of augmin leads to a mixed MT polarity. Interestingly the uniformly retrograde MT polarity in distal dendrites was not affected (Fig. S4). Taken together, these results show that augmin is involved in maintaining a sufficient dendritic MT density *in vivo* and defining the polarity of MTs in neuronal axons.

### Augmin subunits control dendrite elaboration of *Drosophila* c4da neurons *in vivo*

**Fig. 4.**
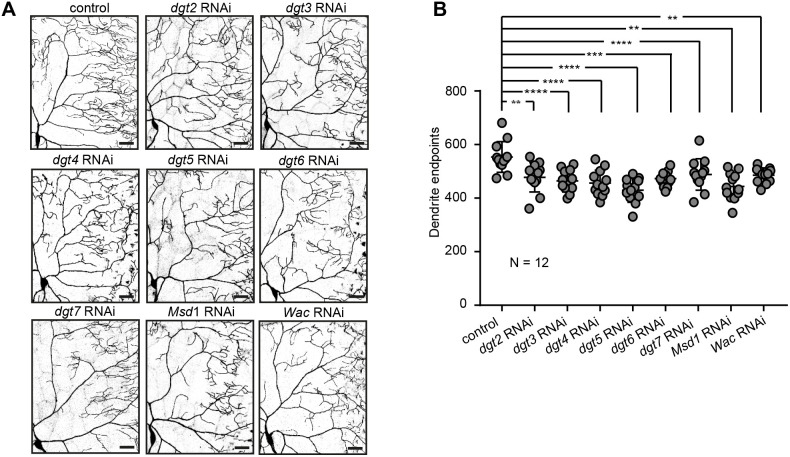
**All augmin complex subunits are required to establish a complex dendrite morphology.** (A,B) Individual *Drosophila* augmin complex subunits were knocked down in c4da neurons of wandering LIII larvae. (A) One quadrant of individual c4da neurons is shown per genotype. Scale bars: 50 µm. (B) The number of dendrite endpoints was reduced upon the knockdown of each augmin complex subunit (mean±s.d.). **P≤0.01; ***P≤0.001; ****P≤0.0001 (one-way ANOVA with Dunnett's post hoc test). *N* reflects number of neurons (biological replicates).

We next tested whether loss of augmin affects the dendritic morphology of the complex c4da neurons. To do so, we depleted the eight individual augmin subunits via RNAi one by one in c4da neurons using *ppk-Gal4* and found a significant reduction in the number of c4da neuron dendritic branches in all cases (Fig. 4A,B). To further confirm the RNAi knockdown result, *dgt5^LE10^* and *dgt6^19A^* mutant alleles were generated by imprecise using P-element excision. The obtained the *dgt5^LE10^* allele is an almost full deletion of the coding region and homozygous mutant *dgt5^LE10^* embryos do not develop into larvae. We also tried to obtain a null mutant *dgt6* allele by mobilization of the {GSV}GS11802 P-element and thereby created the *dgt6^19A^* allele that displayed reduced gene expression. Dgt5 could not be detected in extracts from *dgt5^LE10^* homozygous mutant embryos, supporting that *dgt5^LE10^* is a null mutant, and Dgt6 protein levels were strongly reduced in *dgt6^19A^* third-instar (LIII) mutant larvae extracts, indicating that *dgt6^19A^* is a strong hypomorph (Fig. S2C). In contrast, Dgt5 and Dgt6 proteins could be detected in extracts from mutant animals, carrying *krüppel-Gal4* to drive *UAS-dgt5* or *UAS-dgt6* expression in the embryonic ectoderm (Golembo et al., 1996), respectively (Fig. S2C).

**Fig. 5.**
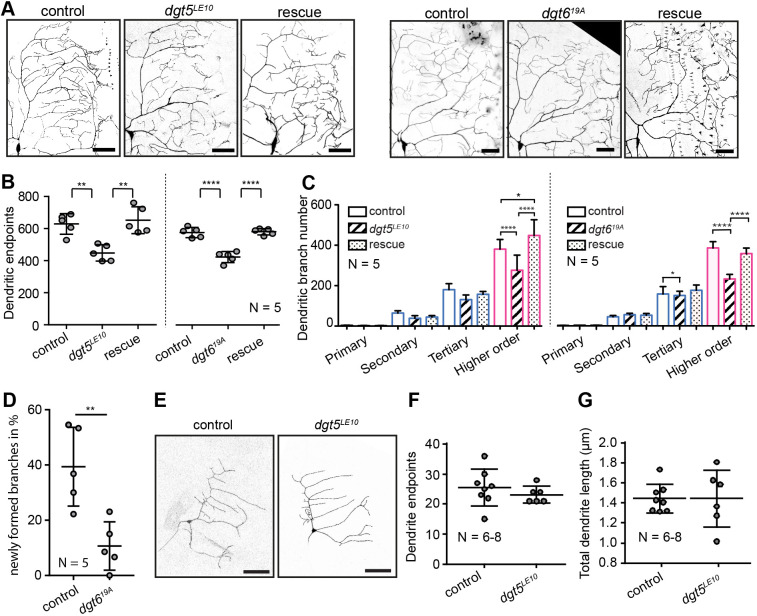
**Dgt5 and Dgt6 regulate terminal dendrite abundance *in vivo* in *Drosophila* c4da neurons.** (A–C) Homozygous mutant *dgt5^LE10^* c4da neurons and mutant *dgt5^LE10^* c4da neurons expressing *UAS-dgt5* (rescue) were obtained in wandering LIII stage using MARCM (Lee and Luo, 2001). c4da neurons were labeled using *Gal4^477^*>*UAS-mCD8::GFP* in wandering LIII larvae, homozygous *dgt6^19A^* mutant animals or *dgt6^19A^* mutants in which *UAS-dgt6* was re-expressed in c4da neurons (rescue). (A) Representative images of a quadrant of the full dendrite tree. (B) Quantifications of neuronal dendritic endpoints. (C) Dendritic branch numbers sorted by branch order. (D) The amount of newly formed branches was measured by *in vivo* time-lapse imaging in control and *dgt6^19A^* mutant neurons at the LII stage. Images were acquired every 5 min for 30 min. (E) c1da control or *dgt5^LE10^* mutant MARCM clones and quantification of their total dendrite length (F) as well as the number of dendrite endpoints (G). Scale bars: 50 µm. All error bars are mean±s.d. *P≤0.05; **P≤0.01; ****P≤0.0001 [one-way ANOVA followed by Tukey's multiple comparisons test (B); two-way ANOVA followed by Tukey's multiple comparisons test (C); unpaired two-tailed Student's *t*-test (D,F,G)]. *N*, number of neurons analyzed.

We next tested whether loss of Dgt5 or Dgt6 function affects dendritic morphology *in vivo* by imaging the dendrites of c4da neurons in immobilized late-stage larvae (wandering LIII stage) using confocal microscopy*.* Given that *dgt5^LE10^* homozygous animals did not survive to the larval stages, homozygous mutant single c4da neuron clones were obtained using the mosaic analysis with a repressible cell marker (MARCM) technique (Lee and Luo, 2001). *dgt5^LE10^* mutant c4da neurons displayed a reduced number of dendrite terminal branches correlating with overall simplified dendrite morphology. A similar simplification of the dendritic arbor was also observed in the c4da neurons of *dgt6^19A^* homozygous mutant larvae at the wandering LIII stage (Fig. 5A,B). To confirm the specificity and to address cell autonomy, we re-expressed wild-type *dgt5* or *dgt6* selectively in the *dgt5^LE10^* or the *dgt6^19A^* mutant c4da neurons, respectively, which largely rescued dendrite morphology (Fig. 5A,B). Augmin depletion affected the MT density in da neurons more in higher-order distal dendrites (Fig. 3B–G). We therefore separately counted the number of primary, secondary tertiary and higher-order dendritic branches in *dgt5^LE10^* or *dgt6^19A^* mutant neurons. This analysis showed that higher-order dendritic branches, including the terminal branches, were reduced in *dgt5^LE10^* or *dgt6^19A^* mutant neurons (Fig. 5C). During development, especially the terminal branches are dynamically growing and retracting (Stürner et al., 2022). To investigate whether loss of augmin function acts on the formation and stabilization of newly emerging branches *in vivo*, we captured the elaboration of c4da neuron dendrites by time-lapse imaging of second-instar larvae [∼72 h after egg laying (AEL)], in which c4da terminal branchlets are highly dynamic. We found a reduction in the number of newly forming branches in homozygous mutant *dgt6^19A^* c4da neurons (Fig. 5D).

C4da neurons display the most complex dendrites among da neurons. Their main branches are enriched in MTs, whereas the higher order branches are enriched in actin (Nithianandam and Chien, 2018; Nanda et al., 2020). By contrast, the simple dendrite branches of c1da neurons all display only a clear microtubule signal (Nanda et al., 2020). We therefore tested whether augmin function is specifically needed for establishing a complex dendritic morphology, such as in c4da neurons, or if neurons with simpler dendritic morphology can also require augmin. To do so, we analyzed the dendrites of *dgt5* mutant c1da MARCM clones. However, in *dgt5^LE10^* null-mutant c1da neurons, dendrite branch numbers and the overall dendrite length were not affected (Fig. 5E–G). We additionally tested c1da neurons in which *dgt5* or *dgt6* expression levels were knocked down via RNAi using the c1da neuron-specific *IG1-Gal4* driver line (Fig. S5). Likewise, dendritic morphology was unaffected in these mutants. Taken together, our data indicate that augmin is cell autonomously required for proper dendrite formation of complex c4da neurons *in vivo.* Given that augmin acts on terminal dendrite formation, we speculated that it might also be localized at the more distal dendritic regions. Dgt6 immunostaining using anti-Dgt6 antibodies was only detectable in c4da neuronal cell bodies and high background signal prevented analyzing potential localization in the dendrites (Fig. S6A). We additionally generated an *UAS-eGFP::dgt6* transgene and expressed it in c4da neurons. This gave a patchy dendritic eGFP::Dgt6 signal that did not localize to any specific dendritic compartments such as branch points (Fig. S6B). Also, the overexpression of this construct significantly reduced dendrite branching, which suggests that this fusion protein acts as a dominant-negative allele (Fig. S6C–E).

### Augmin complex regulates terminal branch dynamics in c4da neurons in cooperation with the γ-TuRC

**Fig. 6.**
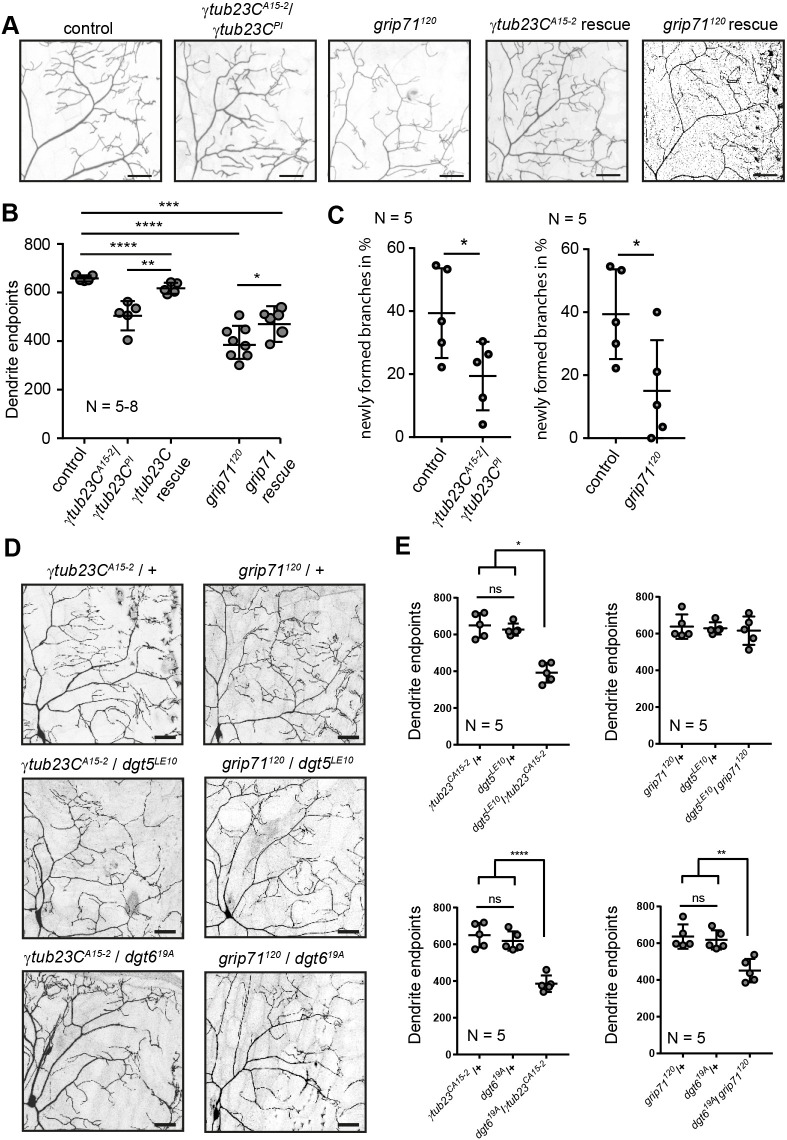
**γ-TuRC and augmin complexes functionally cooperate during dendrite development.** (A) Representative confocal stack images of c4da neurons of a control genotype and the trans-allelic γ*tub23C^A15-2^ /*γ*tub23C^PI^* or *grip71^120^* mutant at wandering LIII stage. Rescue experiments were performed by expression of either *UAS-*γ*tub23C::GFP* or *UAS-grip71::GFP* in c4da neurons using ppk-Gal4 in the respective mutant background. Scale bars: 50 µm. (B) The number of total dendritic endpoints was reduced in γ*tub23C* or *grip71^120^* mutant c4da neurons and could be cell autonomously rescued. (C) The amount of newly formed branches was measured by *in vivo* time-lapse imaging in control, γ*tub23C* or *grip71^120^* mutant c4da neurons at the LII stage. Images were acquired every 5 min for 30 min. (D) c4da neurons of heterozygous γ*tub23C^A15-2^/+*, *grip71^120^/+* larvae or of trans-heterozygous combinations of these γ-TuRC components with heterozygous *dgt5* or *dgt6* mutants. Scale bars: 50 µm. (E) Quantification of total dendritic endpoints reveals a reduction in *dgt5^LE10^/*γ*tub23C^A15-2^*, *dgt6^19A^/*γ*tub23C^A15-2^, and dgt6^19A^/grip71^120^* trans-heterozygous cells but not in the *dgt^5LE10^/grip71^120^* trans-allelic combination compared to single heterozygous mutants. All error bars are mean±s.d. ns, not significant (*P*>0.05); *P≤0.05; **P≤0.01; ***P≤0.001; ****P≤0.0001 [one-way ANOVA with Tukey's post hoc test (B); Kruskal–Wallis test with Dunn's multiple correction test (C); one-way ANOVA with Tukey's post hoc test (E)]. *N* reflects number of neurons (biological replicates).

Augmin is reported to recruit the γ-TuRC to pre-existing MT filaments (Song et al., 2018). We thus tested how loss of γ-TuRC components γTub23C or Grip71 might affect dendrite morphology in c4da neurons in comparison to augmin mutant c4da neurons and found that their absence decreased dendritic complexity (Fig. 6A,B) to a similar extent to that seen with the loss of augmin subunits (Fig. 5A,B). In both cases, dendritic complexity defects could be rescued by re-expression of γTub23C or Grip71 in the respective mutant background in c4da neurons, demonstrating the cell-autonomous function of these subunits on dendrite morphology (Fig. 6A,B).

We next used *in vivo* time-lapse imaging to test whether, similar to what is seen for *dgt6^19A^* mutant neurons, new branch formation is affected in neurons with an impaired γ-TuRC. Although to a milder extent, a lack of γTub23C and Grip71 phenocopied the loss of the augmin subunit Dgt6 (Fig. 5D) by causing a reduction of newly forming branches (Fig. 6C). To test for a potential cooperation between these two complexes, we investigated terminal branch numbers in trans-heterozygous combinations of augmin mutants *dgt5^LE10^* or *dgt6^19A^* with mutations affecting γTuRC components. Whereas heterozygous single mutants of augmin or γTuRC subunits did not display a measurable reduction of dendrite endpoints in c4da neurons, trans-heterozygous combinations of γ*tub23C^A15-2^* with either *dgt5^LE10^* or *dgt6^19A^* mutants or of the *grip71^120^* allele in combination with *dgt6^19A^* yielded branch reduction (Fig. 6D,E) supporting the view that the two protein complexes cooperate to support dendrite formation. Note that the trans-heterozygous combination of *grip71^120^* and *dtg5^LE10^* did not lead to a measurable effect compared to the single heterozygous mutant neurons. Taken together, these results strongly suggest that the augmin complex works together with the γ-TuRC to regulate the dynamics of terminal dendrites in c4da neurons of *Drosophila* larvae.

## DISCUSSION

### Augmin ensures robust MT density in dendrites to drive dendritic development in cultured neurons and *in vivo*

During development, neurons acquire very diverse and cell-type-specific morphologies. This morphological diversity depends on the expression of specific combinations of cytoskeletal regulators that temporally and locally promote a specific branching pattern (Nanda et al., 2020; Stürner et al., 2022). Although a few neuron type-specific actin regulators that determine type-specific morphological characteristics have been uncovered, less is known about neuron type-specific MT nucleation mechanisms (Coles and Bradke, 2015; Kilo et al., 2021; Tavosanis, 2021; Stürner et al., 2022). Multiple lines of evidence together indicate that there is a cooperation between the γ-TuRC and augmin promoting neurite elaboration in cultured hippocampal neurons. In this context, augmin has been suggested to nucleate MTs of the same polarity as the mother MT filament and thereby to increase MT density in a polarity-controlled manner (Sánchez-Huertas et al., 2016; Cunha-Ferreira et al., 2018; Viais et al., 2021).

In particular, depletion of γ-Tub or the augmin subunits HAUS1 or HAUS7 in cultured primary rodent hippocampal neurons at 4DIV leads to a reduction of total α-tubulin levels and subsequently impairs axon specification and outgrowth (Sánchez-Huertas et al., 2016). In more mature neurons (11–12DIV), augmin clusters are distributed along axons and dendrites, suggesting that augmin might also regulate local MT nucleation events in dendrites. Indeed, at this stage HAUS2 or HAUS6 knockdown impairs not only axon but also dendrite outgrowth. Furthermore, this loss of axonal and dendritic growth and complexity is correlated with a reduction in MT density (Cunha-Ferreira et al., 2018). In the present study, we extended those findings to the dendrite organization of primary hippocampal neuron cultures at very early stages (DIV4). Our data show that even at these early stages of differentiation depletion of HAUS1 or HAUS7 impairs dendritic growth and branching by decreasing MT levels, while leaving MT polarity unaffected.

We next examined whether the role of augmin in controlling MT density in dendrites is conserved and relevant *in vivo*. By fluorescently labeling the dendritic MTs of c4da neurons of *Drosophila* larvae, we found diminished levels of MT markers in the distal dendrite segments of augmin-deficient c4da neurons. Additionally, the density of microtubules in thin distal dendrites of da neurons was diminished, as observed in electron microscopy preparations. Together, these data suggest that augmin complex-mediated nucleation is involved in establishing and maintaining dense MT arrays in specific dendritic compartments *in vivo*.

### Loss of augmin does not alter MT polarity in dendrites

In the rodent hippocampal neurons in culture, as well as in *Drosophila* c4da neurons *in vivo*, absence of augmin affects MT polarity in the axon but not in the dendrites – a display of striking conservation (Fig. 2G; Fig. S4). To explain why loss of augmin function does not alter MT polarity in dendrites, we note that the augmin γ-TuRC complex nucleates microtubule branches at shallow angles (Kamasaki et al., 2013; Petry et al., 2013; Verma and Maresca, 2019) and thus branch polarity is determined by the polarity of the mother microtubule. This suggests that augmin does not define *de novo* the orientation of MTs, but that it consolidates their existing orientation. The control of MT orientation in axons might not be very strict; alternatively, a tight control on MT orientation might be temporally restricted to the very early stages of axon formation and elongation. In either case, loss of augmin in axons could lead to some randomized nucleation events without polarity control, which would explain the observed incorrectly oriented (minus-end out) MTs (Sánchez-Huertas et al., 2016; Cunha-Ferreira et al., 2018). By contrast, the mechanisms that define the specific MT orientation in dendrites of different types of neurons might be stricter or these mechanisms could be also kept active in differentiated neurons. Loss of augmin might therefore not lead to polarity defects in dendrites, but merely to a reduction in MT density.

### Loss of augmin function affects the formation of higher-order branches in c4da neurons in cooperation with the γ-TuRC

Using multiple approaches, we showed that the loss of augmin and γ-TuRC affects the formation of higher-order dendritic branches in c4da neurons. Here, loss of augmin function within c4da neurons (Fig. 5A,B) resulted in a similar dendrite simplification phenotype to that seen upon the loss of γ-TuRC activity (Fig. 6A,B). A cooperative function of augmin with the γ-TuRC was further supported by the genetic interaction displayed by subunits of the two complexes (Fig. 6D,E). We therefore suggest that the cooperation with augmin might be an important prerequisite for the γ-TuRC to be recruited and nucleate MTs in distal dendrites to promote formation of higher-order dendrite branches. In line with this suggestion augmin mutant c1da neurons, which have morphologically simple dendrites lacking higher-order branchlets, do not show overall morphological changes (Fig. S5). Interestingly, a parallel study (Mukherjee et al., 2024), published in this issue, has shown similar results and found that compared to what is seen in c1da neurons, augmin expression is increased in c4da neurons and is required in these neurons for the growth of fine dendritic branches.

The above results agree with previous studies showing no overall changes in c1da dendrite morphology when the function of γ-Tub or of the augmin subunit Wac is impaired (Nguyen et al., 2014; Yalgin et al., 2015; Weiner et al., 2020). Interestingly, in c1da neurons the γ-TuRC recruiting factor Centrosomin (Cnn) displays an antagonistic role to Wac (Yalgin et al., 2015). Whereas anterograde polymerizing MTs promote outgrowth and stabilization of nascent dendritic branches (Ori-McKenney et al., 2012; Yalgin et al., 2015), Cnn promotes retrograde MT nucleation and growth from Golgi outposts to restrict dendrite branching in c1da neurons (Yalgin et al., 2015). In c1da neurons, thus, anterograde polymerization promoted by augmin or Wac could support the stabilization of newly formed dendritic branches. The interplay between these two different MT nucleation mechanisms might be a way to regulate dendrite branching by fine-tuning MT polymerization event numbers and MT polarity in c1da neurons (Yalgin et al., 2015; Wilkes and Moore, 2020). It will be interesting to test whether this interplay might also be involved in the distinctive arbor of c4da neurons.

The neuron type-specific variations in phenotype expression between c1da and c4da neurons might be explained by the developmental differences between those two neuronal classes. The main branches of the c1da neuronal dendritic tree are set during the embryonic stage, and afterwards scale with the growth of the animal with only a few new dendritic branchlets being added (Ferreira Castro et al., 2020; Palavalli et al., 2021). By contrast, c4da neurons are continuously gaining dendrite complexity until the late LIII stage (Baltruschat et al., 2020 preprint). This temporal distinction might lead to the availability for c1da neurons of maternally supplied augmin during the phase in which dendrites are established. In contrast, at the time in which higher-order branches are formed in c4da neurons, the augmin level in the mutants might already be neglectable (Vastenhouw et al., 2019). A more intriguing hypothesis would be that different mechanisms are in place in c1da and in c4da neurons to control dendrite branching. Specifically in case of c4da neurons, these would require appropriate MT density guaranteed by augmin function.

Nevertheless, it is important to note that c4da neurons are still capable of forming their type-specific dendritic tree – with just less characteristic space-filling higher-order branchlets. This indicates that the development of higher-order branches in c4da neurons only partially relies on the augmin complex. Indeed, parallel mechanisms of MT nucleation are employed to nucleate MTs and to support dendritic branch formation (Wilkes and Moore, 2020). Early endosomes house Wnt signaling proteins, which recruit γ-Tub to dendritic branch points (Weiner et al., 2020). Furthermore, MTs can even be generated independently of γ-Tub by severing of existing filaments through Katanin and Spastin and transport of short MT fragments into the neurites via motor-based sliding, where they act as local MT nucleation seeds (Yu et al., 2005, 2008; Wood et al., 2006; Stewart et al., 2012). Therefore, all so far described MT nucleation mechanisms might, to a given extent, functionally compensate for the loss of each other. In parallel, *de novo* branchlet formation also strongly depends on actin nucleation (Stürner et al., 2022). Nonetheless, we found no change in localization and abundance of LifeAct–GFP levels in terminal dendrites (Fig. S3G,H). This suggests that, in addition to the above-mentioned MT nucleation mechanisms, actin nucleation could in part compensate for the loss of augmin-dependent MT nucleation and help to maintain a large fraction of terminal dendrites in augmin mutant neurons. How the actin-based and the MT-supported mechanisms might interact in this context is unclear.

Finally, MTs not only provide mechanical support to a cell but simultaneously also function as tracks for MT-based motors to traffic material and machinery needed for neurite branching and growth (Kapitein and Hoogenraad, 2015; Schelski and Bradke, 2017, 2022; Santos et al., 2020). For instance, in axons, reduced γ-Tub levels have been shown to diminish the amount and motility of mitochondria in axons (Sánchez-Huertas et al., 2016). However, to what level transport is affected in augmin-deficient dendrites still needs to be investigated.

In summary, our study establishes the requirement of neuronal augmin function for γ-TuRC-mediated MT nucleation in dendrite development *in vitro* and *in vivo* and, using c4da neurons as a morphologically complex *in vivo* model, reveals a specific role for augmin–γ-TuRC in elaborating the formation of higher-order dendritic branches.

## MATERIALS AND METHODS

### Mice generation and maintenance

To obtain embryonic brain tissue, pregnant 6-week-old female mice (*Mus musculus*; strain OF1) were purchased from Janvier Laboratories and maintained at the animal facilities of the Barcelona Science Park (PCB), in strict accordance with the Spanish and European Union regulations. Protocols were approved by the Animal Care and Use Committee of the PCB (IACUC; CEEA-PCB) in accordance with applicable legislation (Real Decreto 53/2013).

### Hippocampal cell culture

At E17.5–E18.5 days of gestation, females were euthanized by cervical dislocation and embryos were killed by decapitation. Brain tissue was dissected on 10 cm dishes placed on ice containing cold Hank's solution (0.14 M NaCl, 1 mM CaCl_2_, 0.4 mM MgSO_4_·7H_2_O, 0.5 mM MgCl_2_^.^6H_2_O, 0.3 mM Na_2_HPO_4_·2H_2_O, 0.4 mM KH_2_PO_4_, 6 mM D-glucose and 0.4 mM NaHCO_3_, pH 7.4; all reagents from Merck or Sigma). Isolated hippocampi were treated with 0.25% trypsin (Life Technologies) and DNase (Roche) for 15 min at 37°C and dissociated into single cells by gentle pipetting. The neuron density in suspension was calculated by counting cells in a Neubauer chamber. Neurons were seeded on glass coverslips or plastic plates pre-coated with 0.1 mg/ml poly-D-lysine (Sigma) in borate buffer pH 8.5 at 6.6×10^4^ cells per cm^2^ for time-lapse microscopy experiments and at 1×10^4^ cells per cm^2^ for immunofluorescence experiments in plating medium [DMEM containing 10% fetal bovine serum (FBS) and penicillin-streptomycin at 100 IU/ml and 100 µg/ml, respectively]. After 2 h, plating medium was replaced by maintenance medium, consisting of neurobasal medium supplemented with 2% B27, penicillin-streptomycin (100 IU/ml and 100 µg/ml, respectively), 0.6% glucose and glutamax (all reagents from Life Technologies). Neurons were maintained at 37°C in a humidified atmosphere containing 5% CO_2_.

### Lentivirus production and transduction using HEK293T cells

Lentivirus was generated using the LentiLox3.7 system. HEK293T cells (Tim Stearns, The Rockefeller University, USA) were cultured in DMEM containing 10% FBS and penicillin-streptomycin (100 IU/ml and 100 µg/ml, respectively) and kept at 37°C in a humidified atmosphere containing 5% CO_2_. HEK293T cells were co-transfected with a pLKO.1 plasmid containing the desired shRNA (see below), and the viral package and envelope plasmids, by using calcium phosphate. After 72 h, lentivirus particles were concentrated by ultracentrifugation at 27,000 rpm with a SW28 rotor (Beckman) for 2 h at 4°C. Virus particles were resuspended in ice-cold PBS and aliquoted and stored at −80°C. Virus titration was performed by evaluating a GFP-carrying virus produced in parallel with pLKO.1 expressing virus. Infectivity was assayed for GFP-carrying virus by infecting HEK293T cells with serial dilutions of the concentrated lentivirus and sorting of GFP-positive cells by FACS 72 h after infection. Neurons were infected at 1DIV at multiplicity of infection 6. The complete medium was replaced with fresh maintenance medium 16–18 h after infection.

### Immunofluorescence staining of cultured neurons

*In vitro* cultured neurons were fixed at 4 DIV using 4% PFA diluted in PHEM buffer (60 mM PIPES, 25 mM HEPES pH 7.4, 5 mM EGTA, 1 mM MgCl_2_) supplemented with 4% sucrose, 0.25% glutaraldehyde and 0.1% Triton X-100 (Sigma-Aldrich). Fixed cells were washed with PBS and permeabilized with 0.25% Triton X-100 in PBS for 5 min, blocked for 1 h with 4% bovine serum albumin (BSA, Sigma-Aldrich) diluted in PBS and incubated overnight at 4°C with primary antibodies [anti-α-tubulin (DM1A, Sigma, dilution 1:2000) and anti-acetylated-α-tubulin (6-11B-1, Sigma, dilution 1:50,000)], in blocking solution in a wet chamber. Secondary antibodies conjugated to Alexa Fluor 488 or Alexa Fluor 568 were obtained from Life Technologies and used at 1:250.

### Microscopy

To analyze α-tubulin and acetylated-α-tubulin levels, and dendritic and axonal length in HAUS1- and HAUS7-depleted neurons single-plane images were acquired with an Orca AG camera (Hamamatsu) coupled to Leica DMI6000B microscope. For analysis of α-tubulin and acetylated-α-tubulin levels, a 40× objective was used. To assemble mosaics of complete axons and dendritic arbors 20× and 10× objectives were used, and complete mosaics were reconstructed using the LasX software (Leica).

### Microtubule polarity assay

Hippocampal cultures were plated in 0.1 mg/ml poly-D-lysine coated glass-bottom dishes (MatTek), transduced with virus expressing shRNA at 1 DIV and transfected with EB3–Tomato reporter-expressing plasmid at 3DIV. EB3-comets of randomly transfected neurons were imaged 24 h later using an Olympus IX81 microscope equipped with Yokogawa CSU-X1 spinning disc and a temperature-controlled CO_2_ incubation chamber. The proximal dendrite region was defined as ∼20 µm from the soma and distal dendritic region was defined as ∼20 µm away from the dendritic tip. Image stacks were acquired with 100×/1.4 oil immersion objective and an iXon EMCCD Andor DU-897 camera, using iQ2 software. Fluorescence images with a pixel size of 0.14 μm were taken at intervals of 1 s for 150 s. Multiple planes were imaged with a step size of 0.2 μm. *Z*-stacks were acquired by using ImageJ software (NIH; https://imagej.net/ij/).

### EB-1 number and polarity assay

For visualizing polymerizing MTs we used *ppk-EB-1-GFP* flies (Arthur et al., 2015). C4da neurites were identified using *ppk*-mCD4-tdTomato. Flies carrying *ppk-EB1-GFP*, ppk*-mCD4-tdTomato*, and *ppk-Gal4* (generously provided by Sebastian Rumpf, University of Münster, Germany) were crossed to *UAS-Dicer2; UAS-dgt5 RNAi* flies to induce a knockdown. Feeding L3 larvae were mounted in halocarbon oil and immobilized in a specially designed imaging chamber (Dimitrova et al., 2008; Baltruschat et al., 2020 preprint). Image acquisition for the EB1 kymographs was undertaken with a Leica SP8 confocal microscope with a resonant scan head to achieve the needed temporal resolution. Short image stacks of individual c4da neuron dendrite branches and axons, respectively, were recorded with a frequency of one stack per every 2 s for 5 min using a 63× NA 1.4 oil immersion objective. Image stacks were processed and analyzed using ImageJ software. Motion artifacts were corrected using ImageJ 3D drift correction plugin as well as the Image stabilizer plugin (K. Li, https://www.cs.cmu.edu/~kangli/code/Image_Stabilizer.html, February 2008). Statistical data analysis was undertaken using Graphpad Prism [Version 9.1.1.(255)]. EB1 comets in kymographs were also analyzed by a researcher that was not involved in generating the raw data and hence did not know the conditions.

### Image analysis

To measure α-Tub and acetyl-α-Tub signal intensities, images acquired with constant exposure settings and background-subtracted intensities were normalized to the average intensity of the control.

Whole axon and dendritic lengths were measured using the NeuronJ macro (ImageJ software). Sholl analysis was performed using the Sholl analysis plugin as described previously (Ferreira et al., 2014) using binary versions of the dendrite tracings generated with the NeuronJ plugin.

Axonal and dendritic EB3 comet analysis was performed using the kymograph macro (ImageJ software), with lines drawn on the trajectories of comets (Ezquerra et al., 2020).

### Genetics and fly husbandry

Fly stocks and crosses were reared at 25°C on a standard fly medium (corn flour, soy flour, dried yeast, malt, sugar beet treacle; adapted from https://bdsc.indiana.edu/information/recipes/index.html) unless otherwise stated. Generation of *dgt6^19A^*: *dgt6{GSV}GS11802* P-element mutant males were crossed to *yw*; *Pin/CyO*; *Dr Δ{2-3}/TM6*, *Ubx* (Bloomington Stock no. 5908). *dgt6{GSV}GS11802/Dr Δ{2-3}* males of the F1 generation were crossed to a third chromosome balancer line. A white eye phenotype in males of the F2 generation indicated single events of a Δ{2-3} dependent P-element removal and thus the putative generation of a new dgt6 allele trough imprecise P-element immobilization. Stable lines were created by balancing of the third chromosome of single white eyed males over TM6b. Each individual line was tested trough PCR and western blot for deletion of the *dgt6* coding region and *dgt6^19A^* was used for further analysis. Sequencing of the *dgt6^19A^* genomic locus revealed that the protein coding sequence was not affected. However, the mobilization removed the UAS binding sites, which all GSV P-elements contain, and which in our experimental setup would have led to an unwanted Gal4-mediated overexpression of *dgt6*. Generation of *dgt5^LE10^*: *w*; *dgt5^EP2492^* P-element mutant males were crossed to *yw*; *Pin/CyO*; *Dr Δ{2-3}/TM6, Ubx* (Bloomington Stock no. 5908). *Dgt5^EP2492^/Dr Δ{2-3}* males of the F1 generation were crossed to a second chromosome balancer line. Stable lines were created by balancing of the second chromosome of single white eyed males over CyO. Each individual line was tested trough PCR for deletion of the *dgt5* coding region (5′-GCCATCAGGTTGTCCAGCAATTG-3′ and 5′-CCAACTCATCTTCGGAGTCCTC-3′) and *dgt5^LE10^* was used for further analysis. *γtub23C^P1^* was kindly provided by Cayetano Gonzalez (IRB Barcelona, Barcelona, Spain) (Sunkel et al., 1995), *γtub23C^A15-2^* (Vázquez et al., 2008) was obtained from the Bloomington stock center (Nr. 7042), g*rip120* (Reschen et al., 2012) was a kind gift from Jordan W. Raff (University of Oxford, UK), *UAS-γtub23C::GFP* was kindly provided by Melissa Rolls (The Pennsylvania State University, PA, USA; Nguyen et al., 2014).

RNAi lines used were: stock no. 31729 obtained from the Bloomington Stock Center and the following strains were obtained from the VDRC stock center (Vienna, Austria); 60008 (UAS-Dicer2); 103980 (UAS-dgt3 RNAi); 34901(UAS-dgt4 RNAi); 26911 (UAS-dgt5 RNAi); 16352 (UAS-dgt6 RNAi); 50460 (UAS-msd1 RNAi); 21713 (UAS-msd5 RNAi); 104962 (UAS-wac RNAi), ppk-gal4 driven expression of UAS-lacZ was used as a control. The following UAS and gal4 lines used in this study were obtained from the Bloomington Stock Center: 77584 (*w1118; PBac{IT. GAL4}dgt50899-G4/CyO*), 58800 (y1 w*; P{GAL4-Kr.C}10o/TM3, Sb1), 8768 (y1 w*; P{GawB}109(2)80, P{UAS-mCD8::GFP. L}LL5); 8746 (y1 w*; P{GawB}477, P{UAS-mCD8::GFP. L}LL5); 26259 (w[*]; Pin[1]/CyO; P{GawB}221); 55851 (w*; *P{GAL4-da.G32}UH1, Sb^1^/TM6B, Tb^1^*); 32078 and 32079 (*ppk-Gal4*on 2nd and 3rd chromosome). *UAS-lacZ* and *UAS-mCD8::GFP* 3955 (2nd chromosome), 3956 (3rd chromosome); 5131 (2nd chromosome), 5130 (3rd chromosome). *UAS-CD4dtTomato* was a kind gift from Yuh Nung Jan (University of California, San Francisco, USA; Han et al., 2011), *UAS-Jupiter::mcherry* was a gift from Chris Doe (University of Oregon, Oregon, USA; Cabernard and Doe, 2009) and *γTub23C^PI^* was kindly provided by Cayetano Gonzalez (Sunkel et al., 1995). *UAS-LifeAct::GFP* was obtained from the Bloomington stock center (no. 57326).

### Molecular cloning and plasmids

The target sequence for depletion of mouse HAUS7 (shHAUS7: 5′-CCAGATGACCAGGATCTTCTA-3′) and HAUS1 (shHAUS1: 5′-GCTGAACTTACCAAGAAAGTA-3′) were cloned for expression as shRNAs into plKO.1 plasmids. A pLKO.1 plasmid expressing a scrambled sequence (5′-CAACAAGATGAAGAGCACCAA-3′) was used as control. All these pLKO.1 plasmids with shRNA constructs were obtained as bacteria clones from a library of the IRB Barcelona Functional Genomics facility (as a partnership with Sigma-Aldrich RNAi program). The reporter plasmid EB3-Tomato was a generous gift of Anne Straube (University of Warwick, UK).

The following *Drosophila* cDNA clones were obtained from the Drosophila Genomics Resource Center LD47477 (*dgt5*), LD14121 (*dgt6*), RE05579 (*grip71*) and used for the PCR amplification of the coding region using the following primers: 5′-TTAAGAATTCATGAAATGTGCC-3′ and 5′-GATCTCTAGATCATTCTAACAG-3′ (*dgt5*); 5′-CCGGAATTCATGGATCGGACCATAATTGCAC-3′ and 5′-CTAGTCTAGACTAAAAGATAATATCCTTG-3′ (*dgt6*); 5′-TTCCTTTTTTGCGGCCGCATGCATGTT-3′ and 5′-CTAGTCTAGATTACTCTCCGCATGATT-3′ (*grip71*) (Eurofins MWG Operon). PCR amplicons were cloned using XbaI and EcoRI for *dgt5*, EcoRI and XbaI for *dgt6*, and NotI and XbaI for *grip71* into p{UAST}attB (Bischof et al., 2007). The open reading frame of each construct was sequenced. Transgenic flies were generated by BestGene (Chino Hills, US) by PhiC31-mediated integration into landing sites attP 51D (second chromosome) or attP 86F (third chromosome).

The eGFP–Dgt6 expression vector was generated by PCR amplifying *dgt6* cDNA by PCR from the *dgt6* cDNA containing vector (provided by Gohta Goshima, Nagoya University, Japan) using the following primers: dgt6L, 5′-GATCGGACCATAATTGCACCGTGGAAGGCC-3′ and dgt6R, 5′-CTAAAAGATAATATCCTTGAGCACGCTATCGCT-3′. The PCR product was then cloned into the PCR8 vector (Invitrogen), and subsequently was subcloned into Gateway Vectors pTGW (DGRC 1075) for *GFP-dgt6* expression constructs by performing an LR recombination. The construct was microinjected into *w^1118^*;*Δ2-3* (BDSC no. 2534) fly embryos and positive transformants picked upon expression of *mini-white*.

### RT-PCR

The knockdown efficiency for *dgt5*, *dgt6* and *msd1* mRNAs was tested using RT-PCR. *daugthterless (da)-Gal4* (Bloomington Stock no. 8641) was used for the expression (Wodarz et al., 1995) of UAS-dsRNA constructs. Total RNA was extracted from larvae using the RNAzol^®^RT kit (Molecular Research Center, Inc.) and mRNA was reverse transcribed using the ImProm-II™ Reverse Transcription System (Promega) followed by a standard PCR protocol. Primers used were 5′-TAACAGAATTTAAGAACTGGGCCACTAATC-3′ and 5′-TTGTTCTTCAACTCCTGGTCGTAGTTCTT-3′ (*dgt5*); 5′-AACTTCCTGCTCGAGTTCGTGGGCTT-3′ and 5′-ATGGCCTCCTTGAGACCGCACAGAGAT-3′ (*dgt6*); 5′-TGGACAAAATGTTGGCGGGAATGGCG-3′ and 5′-TTCTTCATCTGGCCCACGGTGTCGTA-3′ (*msd1*). Amplification of ribosomal protein L19 cDNA using the following primers 5′-TCTCTAAAGCTCCAGAAGAGGC-3′ and 5′-CGATCTCGTTGATTTCATTGGGA-3′ served as internal control.

### qRT-PCR

RNA was isolated from 10 third-instar larvae using 350 µl lysis buffer using the RNeasy^®^ Mini Kit (Qiagen) following the manufacturer's instructions. Reverse transcription was undertaken using 700 ng RNA and the QuantiTect^®^ Reverse Transcription Kit (Qiagen). The qRT-PCR was undertaken using TaqMan^®^ Probes (Thermo Fisher Scientific) Dm01819973_g1 (cat. no. 4351372) in a qTOWER³ Real-Time-Thermocycler (Analytik Jena).

### Generation of MARCM cell clones

Homozygote *dgt5* mutant c4da neurons were generated using the MARCM technique (Lee and Luo, 2001). To do so, *dgt5^LE10^* was recombined with *FRT42D*. Next, *hsFLP, elav-Gal4 UAS-mCD8::GFP; FRT42D, tubGal80/Cyo* (gift from Takashi Suzuki, Tokyo Institute of Technology, Yokohama, Japan) virgins were collected and crossed to *dgt5^LE10^, FRT42D/CyO-GFP* males. Females were allowed to lay eggs on apple agar plates for 2 h at 25°C and the eggs were allowed to develop for 3 h. Embryos were heat shocked twice 45 min at 38°C in a water bath with a 30 min resting period at room temperature (RT) in between the heat-shock cycles. Plates were kept at 25°C until wandering third-instar larvae could be selected for imaging.

### Western blot analysis

For Dgt5 detection, 20 embryos and for Dgt6 detection five third-instar larvae were selected and grinded in 100 μl 2× Laemmli buffer plus 1 M DTT. Probes were boiled for 5 at 95°C and protein separation by SDS-PAGE was performed under standard conditions. Protein transfer to a nitrocellulose membrane was performed using a Trans-blot Turbo (Bio-Rad). The membrane was blocked with 5% milk powder in washing buffer (0.1% Tween in PBS) for 1 h at RT and was incubated with first antibody in a 1:1000 dilution overnight at 4°C. Anti-Dgt5 antibody was a kind gift from Gohta Goshima's laboratory (Goshima et al., 2008) and anti-Dgt6 antibody was provided by Maria Patrizia Somma's laboratory (Sapienza Universitàdi Roma, Rome, Italy; Bucciarelli et al., 2009). The membrane was washed three times for 5 min each time and detected using HRP-conjugated anti-rabbit IgGs and the ECL detection kit (all from GE Healthcare) in a Chemi-doc (Bio-Rad). Anti-actin antibody was obtained from DSHB Hybridoma (#JLA20).

### Confocal imaging of da neurons and data analysis

Wandering third-instar larvae were embedded in 87–90% glycerol and immobilized in between a glass slide and a cover slip. The dorsal c4da neuron (ddaC) of segment A4 or A5 was imaged using a Zeiss LSM780 or a LSM710 confocal microscope using a 40× NA 1.4 oil immersion objective. Maximum projection images and image adjustments were made using ImageJ and analyzed using the NeuronJ plug-in (Meijering et al., 2004). All dendritic branches were traced and classified into four classes. The long dendrites emerging from the soma were defined as primary dendrite. Dendrites emerged from the primary dendrites were defined as secondary dendrites. Tertiary dendrites were defined as the dendrites emerged from the secondary dendrites and the rest were defined as higher order branches. Each branch was categorized and measured in length.

### Dendritic anti-Futsch signal quantification

A 20 µm wide line was drawn along a labeled (*Gal4109(2)80>UAS-mCD8::GFP*) c4da neuronal dendrite using the region of interest (ROI) manager in ImageJ. The line was straightened and subdivided into 20 bins starting at the soma and reaching out to the dendritic tip. Anti-Futsch labeling (see below) was converted into gray values, background signal intensity subtracted and the average signal intensity calculated for each of the 20 bins. The signal intensity of each bin of a control, UAS-lacZ expressing, dendrites was compared to the corresponding area of dendrites, in which *dgt5* has been knocked down by RNAi.

### Time-lapse imaging

Late second-instar larvae were mounted in halocarbon oil and immobilized in between a metal sieve and a cover slip. One branch of a c4da neuron was imaged under a confocal microscope (Zeiss780) using a 40× NA 1.4 oil immersion objective with an interval of 5 min for 30 min. Stack images were processed with ImageJ software and Photoshop (Adobe) for maximum projections and modifications.

Tracings for each time point were generated using the NeuronJ plug-in (Meijering et al., 2004). The overall number of terminal branches was used to normalize our data set and the amount of newly formed branches was given in as a percentage [(newly formed branches/total branches) ×100]. By definition, we counted a branch as ‘new’ once it appeared for the first time within the 30-min time frame.

### Immunostaining

Open book preparations of wandering third-instar larvae were fixed in 4% formaldehyde with 0.3% Triton X-100 for 20 min at RT. Tissues were washed three times for 10 min each time with PBS plus 0.3% Triton X-100 (PBST). Next, samples were incubated with mouse anti-Futsch antibody (1:100, 22C10, DSHB, Iowa City, USA) primary antibody in PBST plus 10% normal donkey serum (NDS) at 4°C overnight. Afterwards they were washed four times for 10 min each time in PBST and incubated with a Cy5-conjugated anti-mouse-IgG secondary antibody (Jackson ImmunoResearch Laboratories) in PBST plus 10% NDS overnight at 4°C. Preparations were washed 4×10 min in PBST and mounted using PBS with 87.5% glycerol and 0.22 M 1,4-diaza-byciclo (2.2.2) octane (Dabco, Sigma). Confocal stack images of ddaC neurons were obtained using a LSM710 (Zeiss) confocal microscope using a 20× objective.

### Electron microscopy

Sample preparation and detection were conducted as previously described (Tsai et al., 2012). Briefly, dissected larval body walls were fixed at RT for 30 min followed by 4°C overnight in modified Trump's fixative (0.1 M sodium cacodylate buffer, 1% glutaraldehyde and 4% formaldehyde). They were washed three times for 10 min each time in 0.1 M sodium cacodylate, postfixed for 30 min with 2% osmium tetroxide in 0.1 M sodium cacodylate buffer washed three times for 10 min each time with 0.1 M sodium cacodylate buffer and five times for 10 min each time with ddH2O. Specimens were incubated in 2% aqueous uranyl acetate for 20 min, dehydrated by a graded ethanol series, and set into Spurr's embedding medium. Thin sections (90 nm) were stained with uranyl acetate and lead citrate. Images were viewed on a Tecnai G2 Spirit TWIN electron microscope (FEI Company) and captured on a Gatan CCD camera (794.10.BP2 MultiScan). Transmission electron microscopy (TEM) data were quantified by MetaMorph V6.3r7 (Molecular Devices).

### Statistical analysis

Data were analyzed with GraphPad Prism 7 and represent mean±s.d. unless otherwise stated. In box-and-whisker plots, the box represents the 25–75th percentiles, and the median is indicated. The whiskers show the complete range. Asterisks are as follows: ns, not significant (*P*>0.05); *P≤0.05; **P≤0.01; ***P≤0.001; ****P≤0.0001.

## Supplementary Material

10.1242/joces.261512_sup1 Supplementary information
