# Supplementary material for: Augmin complex activity finetunes dendrite morphology through non-centrosomal microtubule nucleation in vivo
Source: J Cell Sci. 2024 May 10;137(9):jcs261512. doi: 10.1242/jcs.261512 (PMC11128282; doi:10.1242/jcs.261512)
Supplement: Supplementary information [file joces-137-261512-s1.pdf]

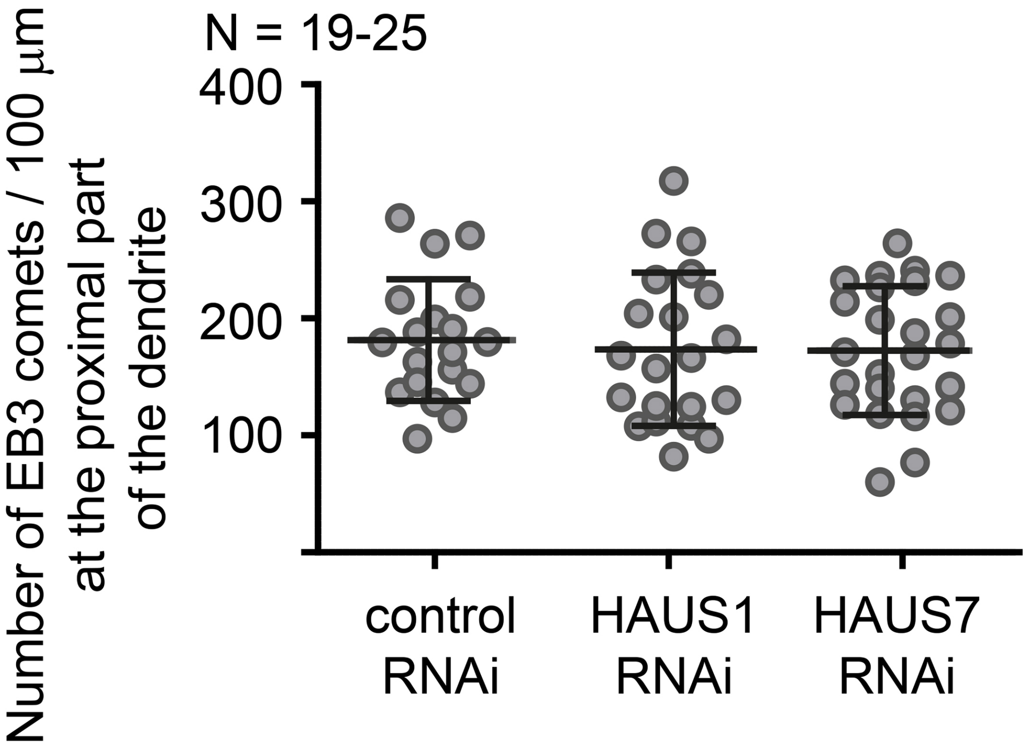

Fig. S1. Number of EB3 comets in proximal dendrites in cultured hippocampal neurons. The number of EB3 comets in primary hippocampal neurons cultures at DIV 5 was unchanged upon HAUS1 or HAUS7 depletion. Statistics: 1-Way-ANOVA with Tukey's *post hoc* test.

Journal of Cell Science • Supplementary information

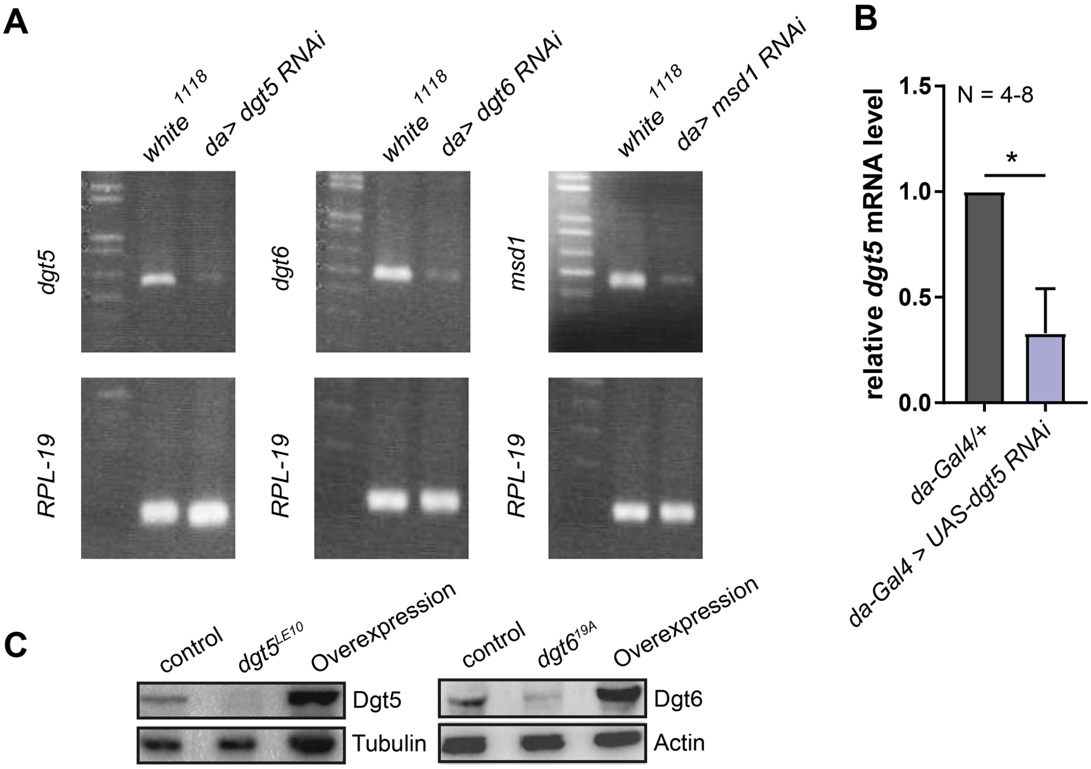

Fig. S2. (A) Knockdown efficiency of *dgt5*, *dgt6* and *msd1* RNAi constructs. Knockdown efficiency was tested by driving the respective UAS-ds RNA construct broadly using daugthterless (da)-Gal4. mRNA was obtained from LIII larvae and amplification of *ribosomal protein L19* (*RPL-19*) was used as control. **(B)** *da-Gal4* was used to drive to test the knockdown efficiency of the *dgt5* RNAi construct. Animals were raised at 27°C. N represents the number of biological replicates. Statistical test used: Mann-Whitney test. **(C)** Western blots loaded with total protein extracts of 20 homozygous mutant embryos (left panel) or five third instar larvae (right panel) and probed with anti-Dgt5/anti-Dgt6 antibodies. α-Tub labeling served as loading control. Dgt5 protein is not detectable in extracts of homozygous *dgt5*^{LE10} embryos; Dgt6 protein levels are drastically reduced in extracts of homozygote *dgt6*^{19A} mutant LIII larvae. Dgt5 and Dgt6 signals could be restored by *krüppel-Gal4*-driven expression of *UAS-dgt5* or *UAS-dgt6* in the respective mutants (rescue).

Journal of Cell Science • Supplementary information

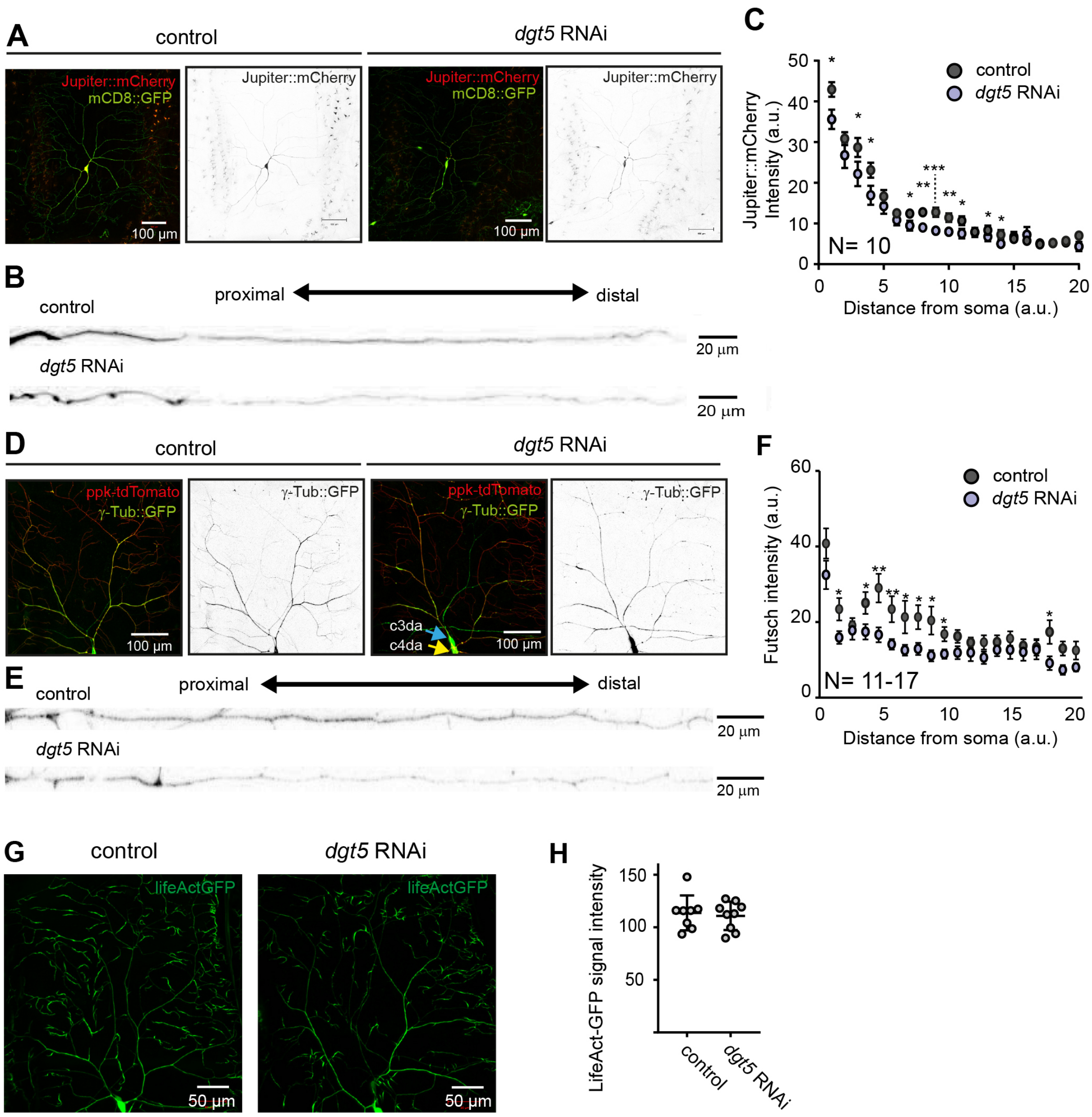

Fig. S3. Dgt5 modulates MT but not actin density. (A-C) C4da neurons expressing the MT marker Jupiter::mCherry and the membrane-associated mCD8::GFP under the control of *ppk-Gal4*. Simultaneously, a control (UAS-lacZ) or a *UAS-dgt5* RNAi construct were co-expressed. **(A)** Representative confocal images. **(B)** Jupiter::mCherry signal along individual dendrites. **(C)** Quantified Jupiter::mCherry signal intensity levels in *dgt5* knockdown and control dendrites**. (D-E)** Control or *dgt5* knockdown c4da neurons labeled by *ppk-tdTomato* expression. Simultaneously-expressed γ-Tub::GFP . **(D)** Representative confocal images. **(E)** γ-Tub::GFP signal along individual dendrites. **(F)** Quantified γ-Tub::GFP signal intensity levels in *dgt5* knockdown and control dendrites**. (G-H)** *UAS-lifeAct* expression was driven by ppk-Gal4 in *dgt5* knockdown and control c4da neurons **(G)** Representative confocal images. **(H)** Quantified LifeAct::GFP levels. Statistics in C, F and H: two tailed student´s *t*-test.

Journal of Cell Science • Supplementary information

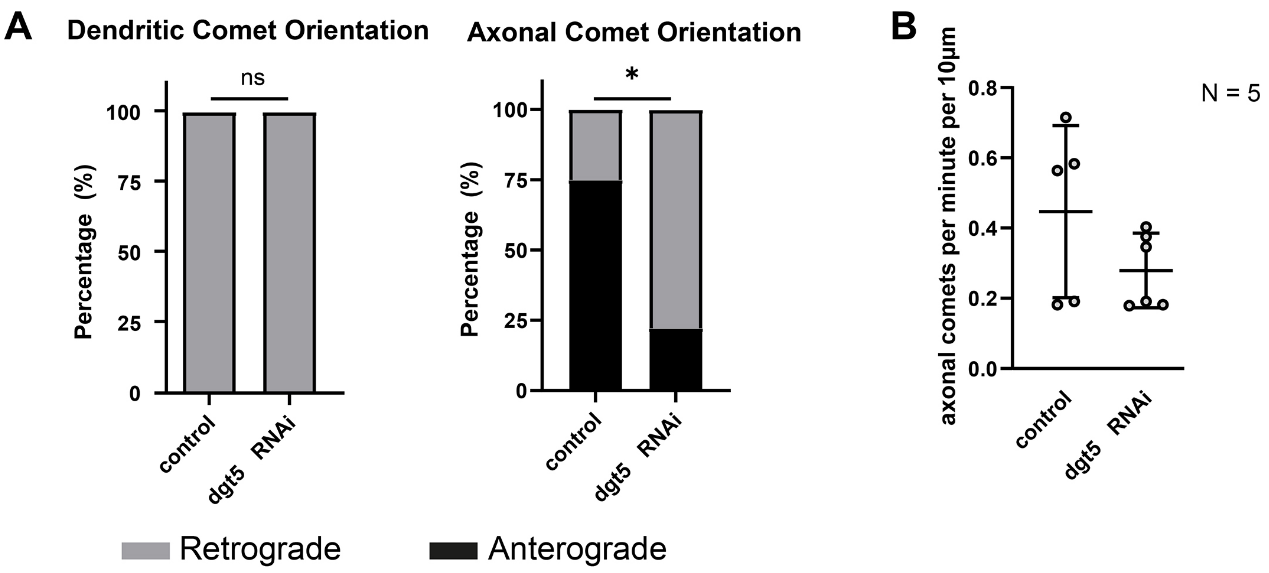

Fig. S4. Dgt5 knockdown affects axonal but not dendritic MT orientation. *ppk*-EB1::GFP comets were imaged in control or dgt5 depleted c4da neurites. **(A-B)** dgt5 RNAi-knockdown affects axonal **(A)** but not dendritic **(B)** MT orientation. **(C)** EB1::GFP comet number was not affected in axons. Statistical test used in C: two-tailed Students *t*-test.

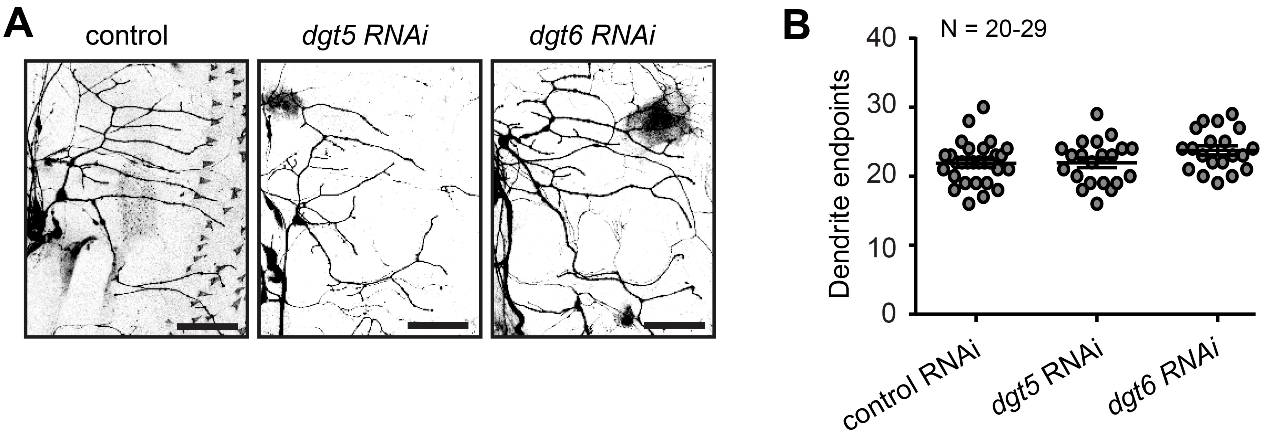

Fig. S5. Reduction of augmin function does not modify c1da dendrite complexity. *UAS-dgt5 RNAi, UAS-dgt6* or a control construct were expressed together with *UAS-mCD8::GFP* under the control of the c1da neuron driver. The number of dendrite endpoints is not modified upon knockdown of *dgt5* or *dgt6*. Scale bars = 50 µm. Statistical test: 1-Way-ANOVA + Dunnett´s *post hoc* test.

Journal of Cell Science • Supplementary information

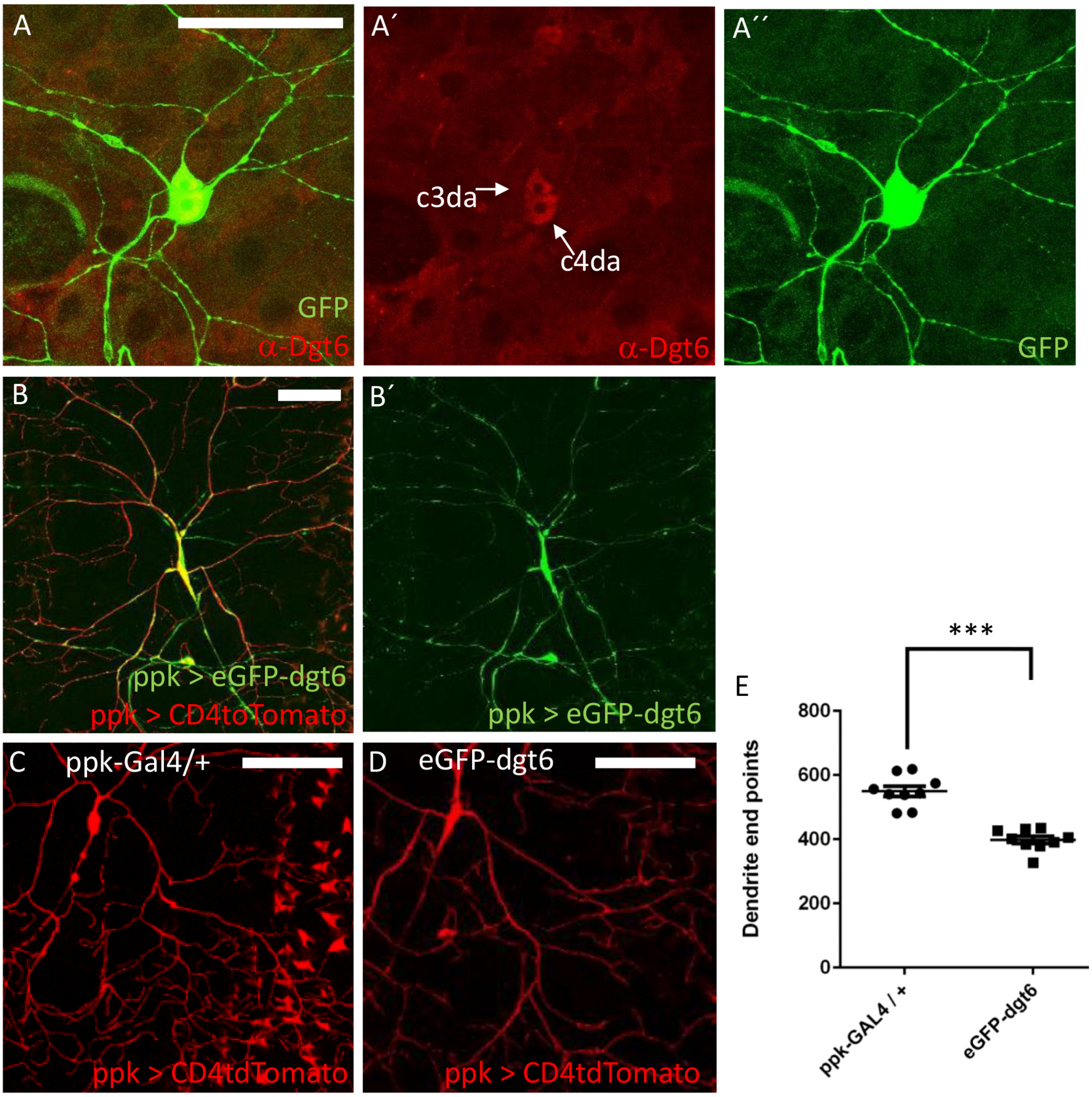

Fig. S6. Dgt6 localization in c4da neurons. (A) Anti-Dgt6 labeling (red) in c4da and c3da neuron labeled by *ppk-Gal4* driven expression of *UAS-mCD8::GFP* (green). **(B-D)** *ppk-Gal4* driven expression of *UAS-CD4tdTomato* was used to label c4da neurons and to simultaneously express *eGFP-dgt6* **(B, D). (E)** The number of dendrite endpoints was reduced upon the expression of *eGFP-dgt6*. Statistical test used in B: two-tailed Students *t*-test. Scale bars = 100 µm.

Journal of Cell Science • Supplementary information
